# *ICOS* Gene Polymorphisms (IVS1 + 173 T/C and c. 1624 C/T) in Primary Sjögren’s Syndrome Patients: Analysis of ICOS Expression

**DOI:** 10.3390/cimb44020053

**Published:** 2022-02-02

**Authors:** José Antonio García-Espinoza, José Francisco Muñoz-Valle, Mariel García-Chagollán, Jorge Hernández-Bello, Claudia Azucena Palafox-Sánchez, Erika Fabiola López-Villalobos, Gabriela Athziri Sánchez-Zuno, Gloria Esther Martínez-Bonilla, Sergio Cerpa-Cruz, Francisco Josue Carrillo-Ballesteros, Edith Oregon-Romero

**Affiliations:** 1 Instituto de Investigación en Ciencias Biomédicas, Centro Universitario de Ciencias de la Salud, Universidad de Guadalajara, Guadalajara 44340, Mexico; antoniojafar25helios@gmail.com (J.A.G.-E.); biologiamolecular@hotmail.com (J.F.M.-V.); maye_999@hotmail.com (M.G.-C.); jorge89_5@hotmail.com (J.H.-B.); kklaumx@yahoo.com (C.A.P.-S.); paliiloa@hotmail.com (E.F.L.-V.); athziri_93_7@hotmail.com (G.A.S.-Z.); 2 Servicio de Reumatología, O.P.D. Hospital Civil de Guadalajara “Fray Antonio Alcalde”, Guadalajara 44280, Mexico; glomarbon@hotmail.com (G.E.M.-B.); sacer04@prodigy.net.mx (S.C.-C.); 3 Departamento de Farmacobiología, Centro Universitarios de Ciencias Exactas e Ingenierias, Universidad de Guadalajara, Guadalajara 44430, Mexico; fjosue_2111@hotmail.com

**Keywords:** ICOS, polymorphism, primary Sjögren’s syndrome, autoimmune diseases

## Abstract

Background: Primary Sjögren’s syndrome (pSS) is a systemic autoimmune disease, which affects exocrine glands. T cell activation is a trigger mechanism in the immune response. Hyperreactivity of T cells and antibody production are features in pSS. ICOS can be critical in the pathogenesis of pSS. Methods: A total of 134 pSS patients and 134 control subjects (CS) were included. Genotyping was performed by PCR-RFLP. *ICOS* mRNA expression was quantified by real-time PCR, and CD4+ ICOS+ T cells were determined by flow cytometry. Results: The *ICOS* IVS1 + 173 T>C polymorphisms were not associated with susceptibility to pSS (*p* = 0.393, CI = 0.503–1.311). However, the c.1624 C>T polymorphism was associated with a reduction in the risk of development of pSS (*p* = 0.015, CI = 0.294–0.884). An increase in *ICOS* mRNA expression in patients was observed (3.7-fold). Furthermore, pSS patients showed an increase in membranal-ICOS expression (mICOS). High expression of mICOS (MFI) was associated with lymphocytic infiltration. Conclusions: The IVS1 + 173 polymorphism is not a genetic marker for the development of pSS, while c.1624 T allele was associated with a low risk. However, elevated mICOS expression in pSS patients with high lymphocytic infiltration was found. ICOS may have an important role in the immunopathogenesis of pSS and should be analyzed in T cell subsets in pSS patients as a possible disease marker.

## 1. Introduction

Primary Sjögren’s syndrome (pSS) is a systemic autoimmune disease characterized by dry eyes and a dry mouth [1], and systemic manifestations, such as general fatigue, fever, and damage to multiple organs [2]. In addition, immunological abnormalities include antinuclear antibodies (ANAs), antibodies directed against Ro or La ribonucleoproteins, and hypergammaglobulinemia [3,4].

In primary Sjögren’s syndrome, an imbalanced immune response is usually mediated by T cells in the early stages of disease [5,6,7], which causes cellular infiltrate. In this line, the expression of co-stimulatory proteins is necessary for the proper functioning of the immune system [8]. Experimental evidence has linked co-stimulatory proteins in many inflammatory processes such as infections, cancer, and autoimmunity [9,10,11]. In general, co-stimulatory molecules can be classified as stimulatory or inhibitory, some are even constitutively expressed such as CD28; however, there are others such as ICOS that are inducible, critical to the T cell response. ICOS results in enhanced signals to activate transcription factors such as nuclear factor-κB (NF-κB), nuclear factor of activated T cells (NFAT), and activator protein 1 (AP1) [12]. In addition, ICOS also directly influences T-helper cell differentiation into T-helper cell type 1 (Th1), Th2, or Th17 subsets [10,12,13], and more recently, ICOS has been directly implicated in the induction of a specific T cell effector subset known as T follicular helper (Tfh) cells [13].

The co-stimulatory receptor ICOS (CD278) is critical for T cell activation and the generation, function, and maintenance of Tfh and extrafollicular T helper cells that help germinal center reaction to produce antibodies [11]. Therefore, *ICOS*-deficient mice and humans almost completely lack this T follicular helper cell subset and have severely impaired humoral response which is essential to resolve the inflammation that occurs in the body [13]. 

However, ICOS has also been analyzed as a regulator of inflammatory T cells in many different disease models. Recent evidence shows that ICOS modulates exacerbated B and T responses in pSS in salivary glands [5,14,15]. For that reason, there is a growing interest in trying to understand the microenvironment in salivary glands (SGs) and how this affects T cell activation to become in a functional cell and generate a chronic cellular response.

Although polymorphisms in *ICOS* are associated with several autoimmune diseases, few studies have investigated the role of ICOS in primary Sjögren’s syndrome. Polymorphisms in *ICOS* have been associated with susceptibility to autoimmune diseases such as coeliac disease [16], pemphigus [17], and autoimmune hepatitis type 1 [18]. Furthermore, recent studies demonstrate that ICOS expression was up regulated in SGs but also in peripheral blood mononuclear cells (PBMCs) in pSS. In addition, the expression of ICOS was closely associated with lymphocytic infiltration in SGs and disease activity of pSS patients [19].

Evidence demonstrated that these polymorphisms (IVS1 + 173 T/C and c.1624 C/T) in the *ICOS* gene affect its transcription [20,21]. This occurs by various mechanisms such as RNA-binding proteins that control gene expression post-transcriptionally by recognizing multiple stem-loop structures in their 3′-UTRs. By this mechanism, Roquin-regulated mRNAs encode costimulatory receptors such as ICOS, CTLA-4, and Ox40 [22]. 

In the present study, given the importance of ICOS as a mediator of inflammation, we analyzed polymorphisms of *ICOS* (IVS1 + 173 T/C and c.1624 C/T) in primary Sjögren’s syndrome which have not been previously studied in this disease. This study aimed to investigate the possible association between *ICOS* polymorphisms and expression in pSS and the severity of the disease. 

## 2. Methods

### 2.1. Study Group

Primary Sjögren’s syndrome patients [(*n* = 134; mean age (range) 55 (29–83); 133 patients were female and 1 was male] who satisfied the criteria of the American College of Rheumatology/European League Against Rheumatism 2016 without any other type of autoimmune diseases, were enrolled from the Rheumatology Service of the Hospital General de Occidente (Zapopan, México) and Hospital Civil Fray Antonio Alcalde, Guadalajara, México. The control subjects’ (CS) mean age was 54 (range: 39–66), including 20 females and 1 male (*n* = 21) for the flow cytometry analysis. Patients and control individuals were native Mexicans living in the Occident of the country (México). This study was conducted by the principles expressed in the Declaration of Helsinki. The participation was voluntary, and all subjects provided written informed consent (CI/037/2016).

The Sjögren’s Syndrome Disease Activity Index (SSDAI), Sjögren’s Syndrome Disease Damage Index (SSDDI), and EULAR Sjögren’s Syndrome Disease Activity Index (ESSDAI) were evaluated in pSS patients. Anti-Ro, anti-La (Orgentec Diagnostika GmbH, Mainz, Germany), antinuclear antibodies (Biomatik, Ontario, ON, Canada), complete blood chemistry (Cell-Dyn 1700, Abbott Laboratories, Abbott Park, IL, USA), erythrocyte sedimentation rate (ESR: performed by Wintrobe’s method), C reactive protein, and rheumatoid factor (by turbidimetry, BS120, Mindray, Shenzhen, China) were measured. The IgG normal range for people older than 19 years of age is between 700–1600 mg/dL and has been adopted for the purpose of this study [23]. The focus score data of minor salivary gland biopsies were obtained from the clinical record of each patient.

### 2.2. Genotype Analysis 

DNA was isolated from peripheral blood by Miller’s modified technique [24]. The polymerase chain reaction and restriction fragment length polymorphism were performed to identify *ICOS* genotypes in 134 patients with pSS and 134 control subjects. Both were recruited from western México; states that comprise this area are Jalisco, Colima, Nayarit, and Michoacán. Polymorphic regions were amplified with the following primers: IVS1 + 173 T>C (rs10932029) forward 5′-CCTCTGGTATTTCTTTCTCTTC-3′, reverse 5′ CCTCTGGTATTTCTTTCTCTTC-3′; c.1624 C>T (rs10932037) forward 5′-CATTATCTATGTTTTCATGGTATT-3′, reverse 5′-AGGCTATCTTGAAGGGCCAG-3′. PCR conditions for IVS1 + 173 T>C polymorphism were the following: a total volume of 25 µL containing 100 ng of gDNA, 1X PCR Buffer, 1.0 mM MgCl_2_, 0.12 µM of each primer, and 0.625 Units of Taq DNA polymerase (Invitrogen, Thermo Scientific, California, USA). Cycling conditions were 1 min of initial denaturalization to 94 °C, 45 s of alignment to 55.8 °C, and 1 min of extension to 72 °C. 5U of *Ddel* enzyme restriction (New England, BioLab, Inc., Ipswich, MA, USA) was used for 30 min to 37 °C; 317 bp represent the wild homozygous genotype (TT); 220 + 97 + 317 bp represent the heterozygous genotype (TC) and 220 bp and 97 bp represent the polymorphic homozygous genotype (CC). For c.1624 C>T (rs10932037) it was the following: a total volume of 25 µL containing 100 ng of gDNA, 1X PCR Buffer, 1.0 mM MgCl_2_, 0.12 µM of each primer, and 0.625 Units of *Taq DNA polymerase* (Invitrogen, Thermo Scientific, CA, USA). Cycling conditions were 1 min of initial denaturalization to 94 °C, 45 s of alignment to 61 °C and 1 min of extension to 72 °C. 5U of *NlaIII* enzyme restriction (New England, BioLab, Inc., Ipswich, MA, USA) was used for 30 min to 37 °C; 270 + 86 + 70 + 19 bp represent the wild homozygous genotype (CC); 270 + 86 +70 + 19 and 156 bp represent the heterozygous genotype (CT) and 270 + 156 + 19 bp represent the polymorphic homozygous genotype (TT). Haplotype analysis was carried out by the SHEsis program.

### 2.3. ICOS mRNA Expression Analysis 

Total RNA was extracted from 5 mL of peripheral blood of 21 pSS patients and 20 CS (matched by age), according to the Chomcyznski and Sacchi technique [25], using TRIzoL reagent (Invitrogen Life Technologies, Carlsbad, CA, USA) according to the manufacturer’s protocol. For mRNA analysis, 1000 ng of total RNA was retro-transcribed using oligo-dT and M-MLV reverse transcriptase as indicated by the manufacturer (Promega, Madison, WI, USA). The quantification of *ICOS* (TaqMan™ Gene Expression Assay, FAM; *Assay IDs*: Hs01057862_m1, Thermo Fisher Scientific, Waltham, MA, USA) mRNA was conducted by real-time PCR, using TaqMan Fast Advanced Master Mix (Applied Biosystems™, Waltham, MA, USA). The glyceraldehyde 3-phosphate dehydrogenase (GAPDH) was used as a reference gene (TaqMan™ Gene Expression Assay, VIC; *Assay IDs*: Hs02786624_g1, Thermo Fisher Scientific, Waltham, MA, USA). All samples were run in duplicate using the conditions indicated in the Gene Expression Assay protocol in a QuantStudio 5 Real-Time PCR Systems (Termo Fisher Scientific, Waltham, MA, USA). The mRNA analysis expression was performed through 2^−ΔΔCt^ after validation of reaction efficiency to determine differences between the study groups.

### 2.4. Flow Cytometry

From peripheral blood samples collected from 21 pSS patients and 20 CS, peripheral blood mononuclear cells (PBMC) were obtained by Ficoll density-gradient centrifugation. PBMC were washed with phosphate-buffered saline. Their viability was evaluated using the trypan blue exclusion method, and only those samples with more than 90% of viability were considered. Multicolor flow cytometry was used to analyze from PBMC. The expression of ICOS on gated CD4+, and CD3+ T cells was analyzed. Cell surface staining was performed with fluorochrome-labeled monoclonal primary antibodies purchased from Biolegend: APC-Cy7 anti-human CD3 (cat: 300426), AF488 anti-human CD4 (cat: 300518), and AF700 anti-human CD278/ICOS (cat: 313528). Corresponding isotype control antibodies were also included from Biolegend. Assay tubes were stained with a mixture of corresponding antibodies at the recommended dilution and incubated for 30 min at room temperature in the dark. After incubation, the cells were washed and fixed. Finally, data were acquired using Attune NxT (Thermo Scientific, Waltham, MA, USA). Data were analyzed with FlowJo software version 10.7 (Becton Dickinson Biosciences Company, NJ, USA).

### 2.5. Statistical Analysis

Statistical analysis was performed by GraphPad Prism 8 (San Diego, CA, USA). The Shapiro–Wilk normality was applied to verify the normal distribution of the data. The Mann–Whitney U test was used for nonparametric data. The Hardy–Weinberg equilibrium was assessed by χ² test. Genotype and allele frequencies were compared by χ² test and *p* values were obtained by Fisher’s Exact Test (frequency < 5%). A *p*-value < 0.05 was considered a statistically significant value. *p* values between data sets were determined by the Mann–Whitney U test or Kruskal–Wallis test.

## 3. Results

### 3.1. Demographic and Clinical Characteristics 

The study includes 134 patients; most of them were female with an average age of 55 years old and 5.62 years of disease duration. Primary Sjögren’s syndrome patients presented a reduction in lacrimal secretion (3.14 mm/5 min) with a moderate lymphocytic infiltration (2.42 foci in 4 mm^2^) (Table 1). Besides ocular and oral sicca manifestations, the patients also presented fatigue (56.4%), arthralgia (59.1%), vasculitis (2.7%), leucopenia (8.2%), peripheral neuropathy (3.7%), and enlargement of parotid gland (3.6%). Patients were treated with prednisone (11.19%), hydroxychloroquine (55.97%), methotrexate (21.64%), and azathioprine (19.40%) (Table 1).

### 3.2. Frequency of ICOS Polymorphisms

Allelic and genotype frequencies of both polymorphisms were evaluated. We found that both polymorphisms were in Hardy–Weinberg’s equilibrium. The distribution of genotypes of IVS1 + 173 *ICOS* polymorphism in patients is presented in Table 2. For RFLP polymorphism analyses (IVS1 + 173 T>C) in patients with pSS, the T allele of polymorphism was found in 86.56% of patients with pSS, compared to 83.95% of control subjects. There was no difference in the allelic or genotypic distribution of IVS1 + 173 between patients and controls.

For patients with pSS and CS polymorphism c.1624 C>T, the C allele was in 91.79% of patients with pSS, compared to 85.07% of controls (Table 2). However, the T allele was associated with a low risk to pSS (OR = 0.510, *p* = 0.015) (Table 2). 

**Figure 1:**
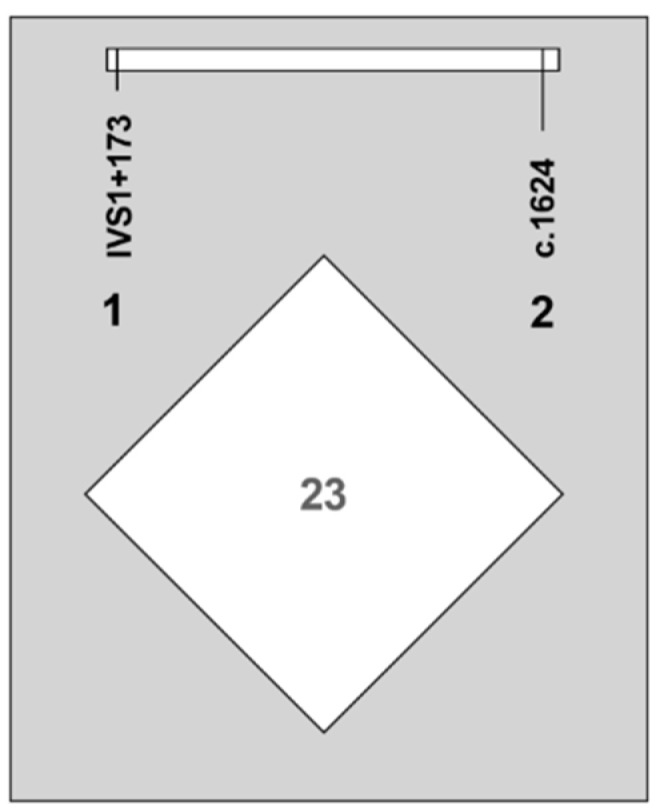
Linkage Disequilibrium of *ICOS*. The haplotype linkage disequilibrium (LD) was calculated with SHEsis program. D’ value of 100 shows a complete LD and a value of 0 shows a complete linkage equilibrium. *ICOS* block shows a low LD (D’ = 23, r^2^ = 0.042).

The linkage disequilibrium (D’) was analyzed with the SHEsis online program for IVS1 + 173 T>C and c.1624 C>T. The *ICOS* polymorphisms showed low LD (D’ = 23, *p* < 0.001, r^2^ = 0.042) (Figure 1). These polymorphisms were not found in a ligand imbalance.

### 3.3. Association of ICOS Polymorphisms and Cellular Expression 

**Figure 2:**
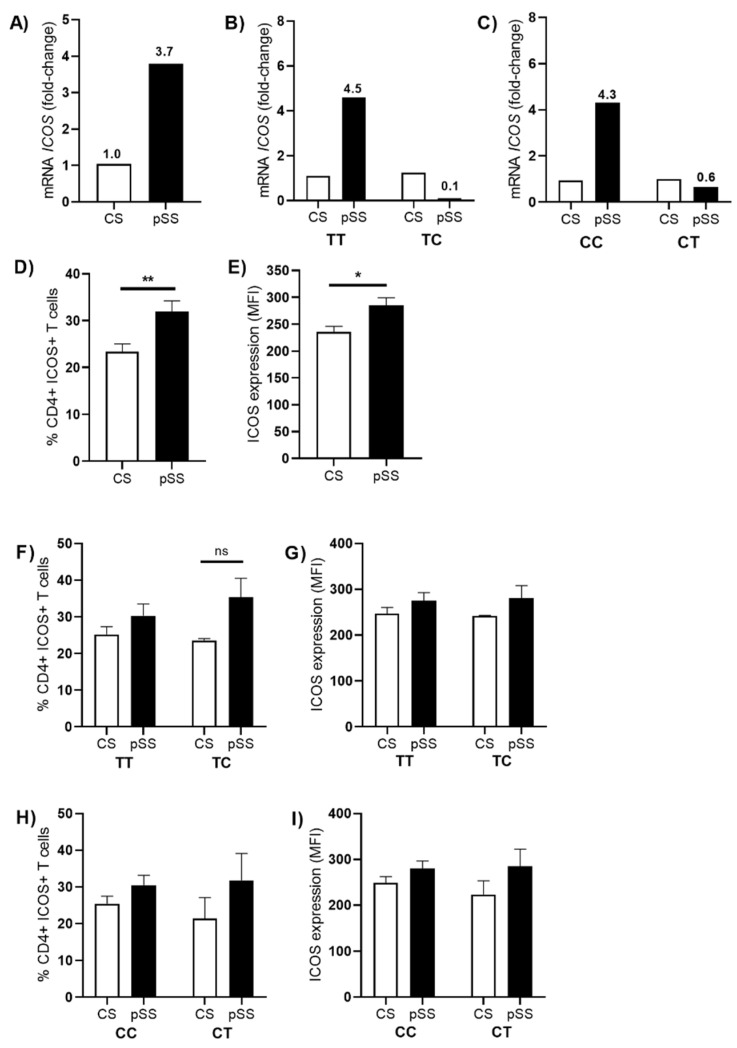
ICOS expression in primary Sjögren’s syndrome. (**A**) Comparison of *ICOS* expression in studied groups. Relative *ICOS* gene expression was determined by the 2^−ΔΔCt^ method using GAPDH as a reference gene. (**B**) Relative expression of *ICOS* according to the polymorphism (IVS1 + 173 T/C) and (**C**) (c.1624 C/T). (**D**) Analysis of T cells in peripheral blood in controls subjects and patients. (**E**) Representation of mean fluorescence intensity (MFI) for ICOS. (**F**) Percentage of CD3+ CD4+ ICOS+ T cells in IVS1 + 173 T/C carriers. (**G**) MFI for ICOS in IVS1 + 173 T/C carriers. (**H**) Percentage of CD3+ CD4+ ICOS+ T cells in c.1624 C/T carriers. (**I**) MFI for ICOS in c.1624 C/T carriers. The level of significance is represented by * *p* < 0.05, ** *p* < 0.01, Mann–Whitney U test, ns = not significative.

The relative mRNA expression of *ICOS* was evaluated in pSS patients and control subjects. Interestingly the relative expression of *ICOS* mRNA was 3.7-fold higher in pSS patients than CS (Figure 2A). However, when separated by genotypes in IVS1 + 173 T/C, compared with the TT genotype (4.5-fold increase), the pSS patient carriers of the CT genotype showed (1.1-fold) less in *ICOS* mRNA than the TT genotype in pSS patients (Figure 2B). Similarly, in c.1624 C/T, we found that the pSS patient carriers of CC had higher *ICOS* mRNA expression than the CT genotype, by 4.3-fold, in CC patients compared with less *ICOS* mRNA in CT patients (1.6-fold), although this was not statistically significant (Figure 2C). 

On the other hand, we were also interested in the percentage of CD4+ ICOS+ T cells in the peripheral blood of patients with pSS. Using the expression of the surface markers ICOS, CD3, and CD4, we categorized T cells into CD3+, CD4+, and ICOS+ as simple positive. Here, there was a significant increase in the frequency of these cells (pSS; 31.30% vs CS; 24.30%, *p* = 0.0157) (Figure 2D). As a result of the increase in CD4+ ICOS+ cells, we evaluate the mean fluorescence intensity (MFI) to measure ICOS between pSS patients and controls. We observed an increase in MFI of ICOS expression (pSS; 264 vs. CS; 242, *p* = 0.0400) (Figure 2E). However, when these data were analyzed by wild-type genotypes and heterozygote genotypes with the percentage of CD3+ CD4+ ICOS+ T cells, we did not find any differences between CS and patients with pSS. In the case of MFI, we did not find any differences either (Figure 2F–I).

### 3.4. Association of ICOS Expression with Focus Score and Immunoglobulins 

**Figure 3:**
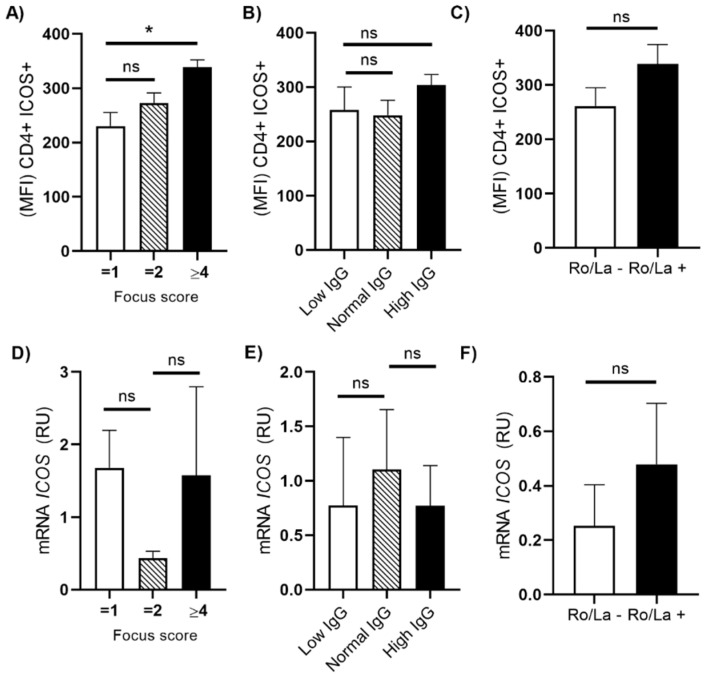
Distribution of ICOS expression in pSS with focus score and antibodies. (**A**) Comparison of ICOS membrane expression (MFI) patients with =1, =2, and 4 ≥ focus score. (**B**) ICOS MFI according to the IgG serum levels (low < 700 mg/dL, normal IgG = 700–1600 mg/dL, and high IgG ≥ 1600 mg/dL). (**C**) (ICOS MFI according to the Ro/La antibodies positive or negative. (**D**) mRNA levels of ICOS according to focus score. (**E**) *ICOS* mRNA expression according to the IgG serum levels. (**F**) mRNA levels of ICOS according to Ro/La antibodies positive. Quantification of mRNA was performed using real-time PCR and the results are expressed as relative units (RU). The level of significance is represented by * *p* < 0.05, Kruskal–Wallis test and Mann–Whitney U-test, ns = not significative.

The ICOS expression related to focus score, immunoglobulin levels, and the presence of Ro/La antibodies as possible risk factors in patients with pSS were evaluated. We observed an increase of ICOS expression (MFI) in patients with ≥ 4 focus [11.67 vs. patients with =1 focus (4.37), *p* = 0.0423, Figure 3A]. However, when these data were analyzed by immunoglobulin levels and Ro/La positivity we did not find any differences between groups (Figure 3B,C). In the case of *ICOS* mRNA expression, we did not find any differences either between groups (Figure 3D–F).

## 4. Discussion

Primary Sjögren’s syndrome is a complex multifactorial disease that involves interactions between genetic and environmental factors for its development [26]. From genome-wide association studies (GWAS) and other studies for Sjögren’s syndrome, some genes have been identified as a potential risk to pSS including *STAT4*, *TNFAIP3*, *IRF5*, *IL12A*, *BLK*, *CXCR5*, and *GTF2I*, and *MHC* alleles [27,28]. However, recent data indicate the importance of ICOS and its interactions between T and B lymphocytes in the development of the autoimmune response [29,30,31]. ICOS modulates the differentiation of antigen-specific B cells into germinal center [13], memory B cell [32], and plasma cells [33]. Moreover, it is reported that ICOS is overexpressed in circulant T follicular helper (cTfh) cells from pSS patients compared to control subjects [34]. 

However, it is unknown how these polymorphisms (IVS1 + 173 T>C and c.1624 C>T) are related to structural effects in the RNA and how these changes affect the RNA binding proteins. For this reason, we used a bioinformatics analysis (data not shown) to evaluate changes in the secondary structure of the RNA polymorphisms, but we did not find any difference between wild-type structure and polymorphisms structure. 

In the present study, we describe the T allele associated with a low risk of developing pSS in the c.1624 C/T polymorphism. In the case of IVS1 + 173 T/C, we did not find any differences. Taking all this into consideration, it suggests that genetic variants in IVS1 + 173 could not be involved in the pathogenic mechanism in pSS. 

In the case of IVS1 + 173, no significant differences were found in the genetic inheritance model’s codominant, dominant, recessive nor in the allelic frequency. The low frequency or absence of polymorphic alleles is probably due to the genetic background of the western Mexican population.

These polymorphisms have been previously identified as genetic markers in hepatitis type 1 and celiac disease in Japanese and Finnish populations [16,18]; also, Hu and colleagues did not find a relation between this polymorphism and hepatitis infection [35]. Nevertheless, there are currently no reports of these polymorphisms in the pSS Mexican population. Haimila and collaborators found [36] that the percentage obtained in the CC genotype for polymorphism IVS1 + 173 was 0%. Similarly, our results showed a 1% CC genotype for polymorphism IVS1 + 173 in the Western Mexican population. These results confirmed that there is no relationship to pSS, nor do they confer protection to the disease.

In addition, LaBerge and collaborators [37], in 126 families from the USA and UK with vitiligo, showed no differences in the frequencies of polymorphism c.1624 where: TT:4, CC: 109, and CT:27, i.e., it had no association with vitiligo; in our case, it also coincides with the frequencies of TT:1, CC:108, and, CT:38 in pSS. 

It is probably for that reason why both polymorphic alleles were found at low frequency in patients (1%) and do not confer a direct relationship with the pSS, due to the European and Amerindian influence within the Mestizo population. Rangel-Villalobos and collaborators [38] explain that the estimated paternal ancestor in the mestizos of western Mexico is mainly European (60–40%), followed by Amerindians (25–21%) and a low percentage of Africans (15%) [38]. However, it was reported that the European influence is high in the north of the country and decreases as the Amerindian influence increases in the central and southern regions in Mexico. 

In the case of polymorphism c.1624, C>T the CC genotype decreases the risk of not rejecting transplantation compared to TT which increases the risk of rejection in kidney transplantation in Finns [16]. This might suggest that the CC genotype in patients with pSS maintains a non-inflammatory profile through ICOS by not promoting direct alteration in pre-mRNA or protein directly. A previous study by Kaartineen et al. [21] showed that on human CD4+ T cells c.1624 CC homozygotes have increased mRNA levels compared with CT and TT individuals. That means the genotype CC is associated with the pathophysiology of pSS [21]. 

A previous report showed that the expression of ICOS on the surface of CD4+ T cells and soluble ICOS in the peripheral blood of patients with systemic sclerosis [39] and systemic lupus erythematosus [40] are significantly increased. In agreement with our results, we observed *ICOS* mRNA levels elevated in patients with pSS (3.7-fold). Primary Sögren’ syndrome patients showed an increase in membranal-ICOS expression (mICOS), which can impact in signaling and differentiation in T cells or participate in germinal center reactions. It is particularly important in the physiopathology of pSS given that T cells require co-stimulatory proteins. However, the expression of the *ICOS* can be affected by other mechanisms such as miRNAs (such as miR-146a on Tfh cells or MiR-27a-3p in CD4+ T cells) [41,42] or RNA-binding proteins [43] that can affect the production of the protein.

This could indicate that ICOS is activated by other immunological mechanisms, such as the secretion of cytokines or other cells. The existence of cytokines, chemokines, and survival factors that modulate susceptibility to pSS is well established. The main cytokines, chemokines, and chemokine receptors in the labial salivary gland are IL-2, IFN-γ, CXCR3, MIP-1α, RANTES, IL-4, IL-10, CCR4, and IL-17 [44]. Other authors have shown BAFF, CXCL13, PD-L2, IL-21, BAFF, IL-21R, and CXCR5 [2,5,45]. Specifically, IL-4, IL-5, IL-10, and IL-13 induce ICOS expression and exhibit potent inflammatory effects in vivo [46]. In addition, binding ICOS to ICOS-ligand (ICOS-L) activates a cascade of intracellular signaling molecules that lead to the production of cytokines such as IL-4 and IFN-γ [47]. 

It has been suggested that ICOS is involved in the differentiation of CD4 T cells. Mahajan et al. [33] demonstrate that ICOS −/− CD4 T cells do not migrate into B cell follicles and do not help wild-type B cells to produce antibodies, indeed they demonstrated that ICOS is important to produce all IgG subclasses [33]. However, our data showed that in both polymorphisms there was no correlation with percentage CD3+ CD4+ ICOS+ T cells. 

Another important finding of this study was a higher membrane expression of ICOS (mICOS) on T cells from the PBMCs of patients with primary Sjögren syndrome. This result was also supported by Luo and collaborators, where expression levels of ICOS were higher in transcriptomic studies in patients with pSS. All these results showed that ICOS was closely associated with typical manifestations of pSS (focus score, anti-Ro/La positive groups, and high serum IgG groups) [19]. We further confirmed that the high expression of mICOS (MFI) was closely associated with lymphocytic infiltration (focus score) equal to or higher than 4. In addition, the threshold of ≥3 foci has a positive predictive value for lymphoma [48].

Nevertheless, it has been described that CD28, ICOS, and CD40 are necessary for the generation of pathogenic Th1/Th17 cells after stimulation [49,50]. We supposed that patients have distinctive clinical manifestations and worse prognosis that require extensive treatments, related to the high capacity of these cells to differentiate to Th17 cells or other subsets included circulant T follicular helper cells (cTfh). This would suggest that CD4+ ICOS+ T cells could promote either germinal center or effector Th1/Th17 cells under inflammatory conditions. 

To the best of our knowledge, there is no evidence of alteration in ICOS in patients treated with hydroxychloroquine and azathioprine. With respect to methotrexate, in rheumatoid arthritis (RA) patients treated with methotrexate [RA(MTX)] or tumor necrosis factor (TNF)-α inhibitors [RA(TNFi)], the expression of ICOS by CD8+CD28− Treg was found to be significantly lower in both RA(MTX) and RA(TNFi) when compared with healthy subjects [51].

In the case of the corticosteroid effect, there are some reports in T follicular helper cells or circulant T follicular helper cells which display contrasting results. In myasthenia gravis (MG) patients, Bortone et al. found that ICOS were expressed at higher levels in Germinal Centers of both untreated and corticosteroid-treated patients than in control thymuses [52]. On the other hand, in IgA nephropathy (IgAN) patients, Wang et al. showed that corticosteroid treatment of patients with IgAN significantly reduced the percentage of circulating ICOS+PD-1hiCXCR5− T cells (cTFh cells) [53].

Future studies in a larger number of samples are needed to investigate both polymorphisms but it is important to study ICOS characterization in different T cell populations to elucidate its contribution to the damage in primary Sjögren’s patients.

In summary, the IVS1 + 173 polymorphism is not a genetic marker for the development of pSS, while the c.1624 T allele was associated with a low risk. However, elevated membrane ICOS expression in pSS patients with high lymphocytic infiltration was found. ICOS may have an important role in the immunopathogenesis of pSS and should be analyzed in T cell subsets in pSS patients as a possible disease marker.

## Figures and Tables

**Table 1:** Demographic and clinical characteristics in Primary.

Sjögren’s Syndrome Patients (pSS)	
Features	*pSS (n = 134)*
Demographics	
Age, years (range)	55 (29–83)
Sex (F/M)	133/1
Disease duration (years)	5.62 ± 4.51
Inflammation markers	
CRP (mg/L)	3.73 ± 3.02 (0.20–24.40)
ESR (mm/h)	26.89 ± 15.10 (0–76)
Clinical parameters	
Schirmer ≤ 5 mm/5 min	3.14 ± 2.12 (0–17)
Foci number ≥ 1 focus/4 mm^2^	2.42 ± 1.28 (0.5–8.0)
SSDAI score (Min–Max)	2.12 ± 1.63 (0–6)
SSDDI score (Min–Max)	1.35 ± 1.05 (0–5)
ESSDAI score (Min–Max)	3.15 ± 3.89 (0–19)
Anti-Ro UI/mL (%)	32.24 ± 58.96 (31.34)
Anti-La UI/mL (%)	12.42 ± 25.09 (12.68)
ANA, *n* (%)	64 (47.76)
FR positive UI/mL (%)	32.06 ± 34.55 (53.73)
Treatment *	
Prednisone, *n* (%)	15 (11.19)
Hydroxychloroquine, *n* (%)	75 (55.97)
Azathioprine, *n* (%)	26 (19.40)
Methotrexate, *n* (%)	29 (21.64)

Data provided on average (minimum and maximum). Accounts; ESR; erythrocyte sedimentation rate, FR; rheumatoid factor, SSDAI; Sjogren’s Disease Activity Rate, SSDDI; Sjogren’s Disease Damage Index, ESSDAI; the activity rate of EULAR Sjogren’s syndrome disease. * Treatment include monotherapy and polytherapy with immunosuppressors/immunomodulators drugs.

**Table 2:** *ICOS* allelic and genotype frequencies. Observed and expected frequencies in all polymorphic sites were in Hardy–Weinberg equilibrium. Significant *p* values are shown in bold. OR (odds ratio), CI (confidence interval), pSS (primary Sjogren Syndrome), CS (control subjects). The *p*-value was calculated by a Chi-squared (χ^2^).

IVS1 + 173 T/C and c.1624 C/T Genotypic and Allelic Frequencies					
	Genotype	CS (*n* = 134)% (*n*) Controls	pSS (*n* = 134)% (*n*) Cases	*p* Value	OR (CI 95%); *p*
IVS1 + 173 T>C (rs10932029)					
Codominant	TT TC CC	69.40 (93) 29.11 (39) 1.49 (2)	73.88 (99) 25.37 (34) 1.74 (1)	0.499	1 0.819 (0.477–1.405); 0.468 0.70 (0.042–5.267); 0.530
Dominant	TT TC + CC	60.40 (93) 30.59 (41)	73.88 (99) 26.11 (35)	0.416	1 0.802 (0.471–1.366); 0.416
Recessive	TT + TC CC	98.50 (132) 1.49 (2)	99.25 (133) 0.75 (1)	0.561	1 0.496 (0.044–5.539); 0.561
Alleles	T C	83.95(225) 16.04 (43)	86.56 (232) 13.43 (36)	0.393	1 0.812 (0.503–1.311); 0.393
**c.1624 C>T** **(rs10932037)**					
Codominant	CC CT TT	70.89 (95) 28.35 (38) 0.74 (1)	85.32 (113) 14.92 (20) 0.74 (1)	0.455	1 0.442 (0.241–0.811); 0.007 0.841 (0.052–13.62); 0.902
Dominant	CC CT + TT	70.89 (95) 29.10 (39)	84.32 (113) 15.67 (21)	0.008	1 0.453 (0.249–0.822); 0.008
Recessive	CC + CT TT	99.25 (133) 0.75 (1)	99.25 (133) 0.75 (1)	1.00	1 1.0 (0.062–16.155); 1.000
Alleles	C T	85.07 (228) 14.92 (40)	91.79 (246) 8.20 (22)	0.015	1 0.510 (0.294–0.884); 0.015

## Data Availability

The data presented in this study are not publicly available.
